# Irisin Modulates Inflammatory, Angiogenic, and Osteogenic Factors during Fracture Healing

**DOI:** 10.3390/ijms24031809

**Published:** 2023-01-17

**Authors:** Angela Oranger, Roberta Zerlotin, Cinzia Buccoliero, Lorenzo Sanesi, Giuseppina Storlino, Ernestina Schipani, Kenneth Michael Kozloff, Giorgio Mori, Graziana Colaianni, Silvia Colucci, Maria Grano

**Affiliations:** 1 Department of Precision and Regenerative Medicine and Ionian Area, University of Bari, 70124 Bari, Italy; 2 Department of Biosciences, Biotechnology and Environment, University of Bari, 70124 Bari, Italy; 3 Department of Translational Biomedicine and Neuroscience, University of Bari, 70124 Bari, Italy; 4 Department of Orthopaedic Surgery, University of Pennsylvania, Philadelphia, PA 19107, USA; 5 Departments of Orthopedic Surgery and Biomedical Engineering, University of Michigan, Ann Arbor, MI 48109, USA; 6 Department of Clinical and Experimental Medicine, University of Foggia, 71122 Foggia, Italy

**Keywords:** fracture, irisin, TNFα, MIP-1α, VEGF, MMP-13, BMP2

## Abstract

Bone fractures are a widespread clinical event due to accidental falls and trauma or bone fragility; they also occur in association with various diseases and are common with aging. In the search for new therapeutic strategies, a crucial link between irisin and bone fractures has recently emerged. To explore this issue, we subjected 8-week-old C57BL/6 male mice to tibial fracture, and then we treated them with intra-peritoneal injection of r-Irisin (100 µg/kg/weekly) or vehicle as control. At day 10 post fracture, histological analysis showed a significant reduced expression of inflammatory cytokines as tumor necrosis factor-alpha (TNFα) (*p* = 0.004) and macrophage inflammatory protein-alpha (MIP-1α) (*p* = 0.015) in the cartilaginous callus of irisin-treated mice compared to controls, supporting irisin’s anti-inflammatory role. We also found increased expressions of the pro-angiogenic molecule vascular endothelial growth factor (VEGF) (*p* = 0.002) and the metalloproteinase MMP-13 (*p* = 0.0006) in the irisin-treated mice compared to the vehicle ones, suggesting a myokine involvement in angiogenesis and cartilage matrix degradation processes. Moreover, the bone morphogenetic protein (BMP2) expression was also upregulated (*p* = 0.002). Taken together, our findings suggest that irisin can contribute to fracture repair by reducing inflammation and promoting vessel invasion, matrix degradation, and bone formation, supporting its possible role as a novel molecule for fracture treatment.

## 1. Introduction

Fractures are common traumatic injuries in humans. As life expectancy advances, the incidence of bone fractures in the world population is dramatically increasing, as are the healthcare costs associated with the occurrence of fractures.

Bones have a high regenerative capacity in mammals, and thus the healing of fractures usually occurs spontaneously. However, in older subjects or patients suffering vascular or metabolic disorders, surgery [1] and/or pharmacological treatments [2] are necessary to promote and/or improve fracture healing.

Endochondral ossification represents an important event involved in fracture healing. It consists of a complex repair process involving stem cells that differentiate into chondrocytes responsible for the formation of a soft callus that is later replaced by bone [3]. It is a multi-step process requiring months to be completed, traditionally divided into four steps: (1) inflammation and mesenchymal stem cell requirement, (2) soft callus formation and revascularization, (3) hard callus formation, and (4) bone remodeling [3].

Phase 1 occurs immediately following the injury: a hematoma is formed and inflammatory events, consisting of both immune cell requirements and proinflammatory molecule secretion, occur. The cytokines and growth factors released during this first step are strongly involved in the cellular and molecular events that characterize fracture healing. Among these molecules are the macrophage inflammatory protein-alpha (MIP-1α) [4] and, importantly, tumor necrosis factor-alpha (TNFα), whose concentration has been demonstrated to peak at 24 h and to return to baseline values after 72 h post injury [5]. Elevated TNFα concentration is essential for initiating the requirement of mesenchymal stem cells with chondrogenic and osteogenic potential from bone marrow, blood, periosteum, and bone cortex [6,7]. It has been shown that TNFα increased proliferation and migration of rat bone marrow mesenchymal cells in vitro. In agreement, blocking TNFα reduced mesenchymal infiltrate and cell proliferation on day 8 in the injured growth plate in vivo. However, in parallel, expression of osteocalcin and trabecular bone formation at the injury site increased, suggesting that the amount of TNFα signaling could be highly regulated at the injury site to achieve the desired effect [8].

Thus, a few days after fracture, mesenchymal progenitor cells, attracted by inflammatory cytokines reach the fibrin-rich granulation tissue and differentiate into chondrocytes. Thus, phase 2 of endochondral ossification, characterized by the formation of a soft callus between the fracture ends, occurs [9]. Crucial for this phase, during which the soft tissue gives the fracture a stable structure, is the bone morphogenetic protein 2 (BMP2), since mice with inactivating mutations in this molecule are unable to form calluses after fracture [10]. It has been shown that the absence of BMP2 in mice inhibits the differentiation of mesenchymal progenitor into chondrocytes or osteoblasts at the fracture site, thus identifying BMP2 as an essential mediator for fracture repair [10]. During fracture healing, the cartilage-to-bone transition is necessary to obtain rigidity and stability. Essential for this transition is the differentiation of chondrocytes into hypertrophic ones, expressing matrix metalloproteinase 13 (MMP-13), which is necessary for cartilage degradation [11,12], and vascular endothelial growth factor (VEGF), which is required for angiogenesis into the avascular cartilage region. In particular, MMP-13 deficiency causes a delay in cartilage resorption in the callus due to impaired resorption of the cartilaginous substrate such as aggrecan [12,13]. Furthermore, VEGF plays an essential role in fracture repair as blockade of its receptors inhibits the regenerative process, while its administration promotes fracture healing [14].

Vascularization represents a key event in both the soft-to-hard callus transition (phase 3 of fracture healing) and in bone remodeling (phase 4 of fracture healing) as it results in the migration of osteoclast and osteoprogenitor precursors.

Muscle tissue is closely associated with bone, and when it is damaged following a fracture, the complication rate of fracture healing is higher [14]. Recently, much evidence showed that skeletal muscle produces numerous myokines having hormone-like actions on bone tissue. Among these myokines, there is irisin, a recently discovered molecule produced in high quantity during physical exercise in both mice and humans. It is initially synthesized as a membrane-linked protein precursor, fibronectin type III domain-containing protein 5 (FNDC5), whose extracellular domain undergoes proteolytic cleavage becoming a small peptide released into the bloodstream.

Numerous scientific studies demonstrated that irisin has an anabolic effect on bone, both in vitro and in vivo [15]. In vitro experiments demonstrated that irisin treatment enhanced osteoblast differentiation [16] and inhibited osteocyte apoptosis [17]. However, irisin’s in vitro effects on osteoclasts are controversial. In particular, Estell et al. provided evidence that irisin promotes osteoclastogenesis [18], while Zhang et al. demonstrated the inhibitory effect of irisin on osteoclast formation [19]. These opposite results are probably due to temporal differences, with a short (7 days) or long (2 months) and chronic duration of irisin treatment by inhibiting or promoting osteoclastogenesis, respectively. In addition, other authors demonstrated that irisin promotes proliferation but inhibits differentiation in osteoclast precursors [20]. Recently, studies performed in microgravity conditions demonstrated that the myokine promoted osteoblast differentiation and activity and indirectly inhibited osteoclastogenesis by increasing osteprotegerin (OPG) expression [21]. Moreover, irisin increased bone mass in healthy mice [22] and, in hindlimb unloaded mice, a well-known osteo-sarcopenic mouse model of disuse-induced osteoporosis and muscle atrophy, irisin promotes osteogenesis, increases osteocyte viability, and prevents disuse-induced loss of bone and muscle mass [17,23].

On the contrary, the study of Estell et al. demonstrated that chronically forced expression of the irisin precursor Fndc5 leads to reduced cortical thickness and trabecular bone volume in young male transgenic mice [18]. The apparently contradictory result is probably due to overexpression of Fndc5/irisin, which is not comparable to the dosage and timing of administered exogenous irisin used in the previous studies.

In addition to in vivo and in vitro studies, clinical works have also provided evidence for the positive role of irisin in bone tissue, although skepticism has been expressed about the quality of some commercially available ELISA (enzyme-linked immunosorbent assay) kits used to measure circulating irisin levels [24], principally due to possible cross-reactivity between the polyclonal antibodies and non-specific proteins in human and animal sera [25]. The existence of irisin itself in humans has been in the spotlight of contradictory observations. However, to date, through the parallel use of ELISA and quantitative mass spectrometry, several sensitive approaches have been found to be successful in confirming the identity of irisin and measuring its circulating level in humans [26]. Human studies have shown that irisin levels are low in postmenopausal women with osteoporotic fractures [27] and are inversely correlated to vertebral fragility fractures [28]. Recently, it has also been demonstrated that low concentrations of irisin in older women are associated with an increased risk of hip fractures [29]. Moreover, a positive correlation between irisin serum levels and bone mineral status in adult and childhood populations has been observed [30,31]. Recently, it has also been demonstrated that cartilage represents another irisin target, since the myokine directly affects human chondrocytes by improving their anabolism while decreasing catabolism [32]. Additionally, irisin delayed osteoarthritis development by protecting inflamed chondrocytes against apoptosis, oxidative damage, and extracellular matrix underproduction [33].

Recently, we demonstrated that pharmacological treatment with irisin accelerates the fracture healing process in mice [34]. In particular, we showed that irisin promotes bone formation at day 10 post fracture and increases total volume, bone volume, and bone mineral content at day 28 post injury, thus accelerating the overall fracture repair process. In agreement with this study, other authors also showed irisin ability in promoting fracture healing by stimulating osteogenesis and angiogenesis [35].

Thus, in order to better understand irisin involvement in mediating fracture healing, we studied its effects in modulating markers of inflammation, angiogenesis, matrix degradation, and bone formation in fractured mice at day 10 post fracture.

The data obtained bring new insights into the important relationship between irisin and inflammation, which has only recently emerged. Furthermore, we provide evidence for a possible involvement of irisin in the modulation of MMP-13, which has never been investigated before and is implicated in the degradation of the cartilage matrix in the callus.

## 2. Results

### 2.1. Irisin Increases Soft Callus Area at 10 Days Post-Fracture

**Figure 1:**
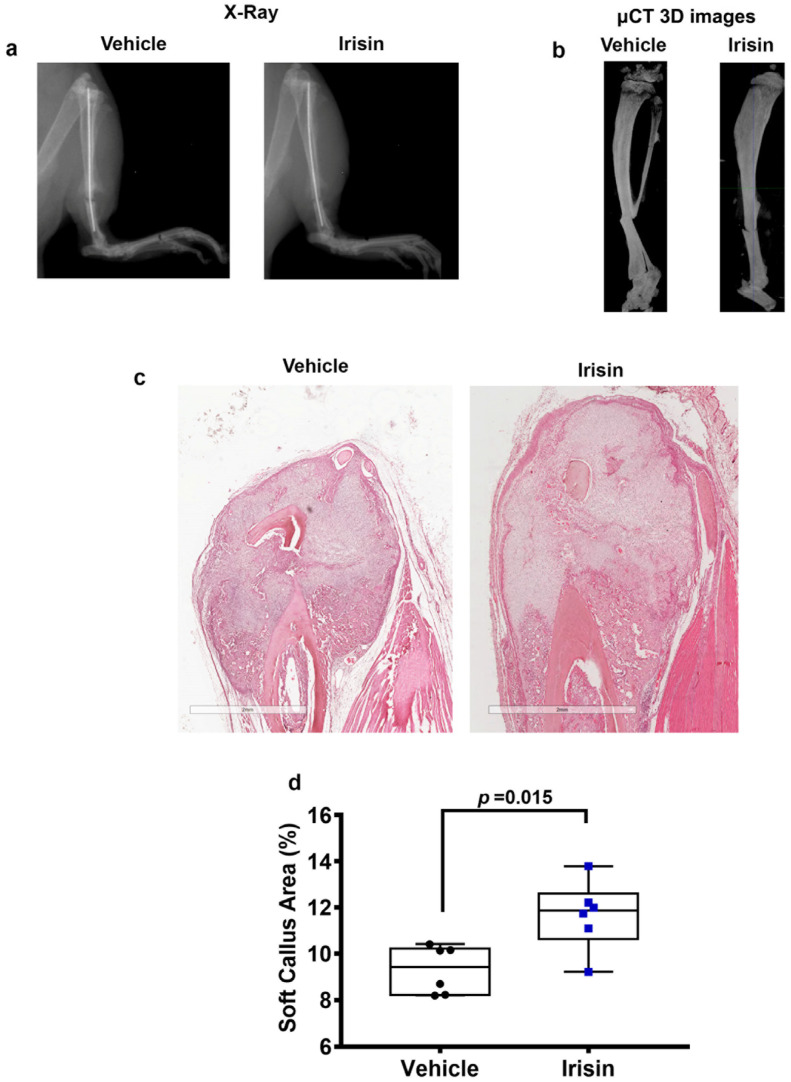
Representative images of (**a**) X-rays and (**b**) longitudinal sectional micro-computed tomography (μCT) 3D reconstructions of fractured tibiae from vehicle-treated mice and irisin-treated mice at 10 days after fracture. Representative H&E images on callus section from vehicle- and irisin-treated mice at 10 days after fracture (scale bar 2 mm) (**c**). Dot plots showing increased soft callus area in irisin-treated mice (*n* = 6) compared with vehicle-treated mice (*n* = 6) (**d**).

X-ray radiography and longitudinal micro-computed tomography 3D reconstructions of tibiae from representative mice, treated with vehicle or irisin at days 0 and day 7, showed clearly visible fracture lines in both treatment groups at 10 days post fracture (Figure 1a,b). To determine the callus area, hematoxylin and eosin (H&E) staining was performed (Figure 1c). Histological analysis revealed increased soft callus area in irisin-treated mice (Figure 1d).

### 2.2. Irisin Induces Inhibition of Inflammation Markers at 10 Days Post Fracture

**Figure 2:**
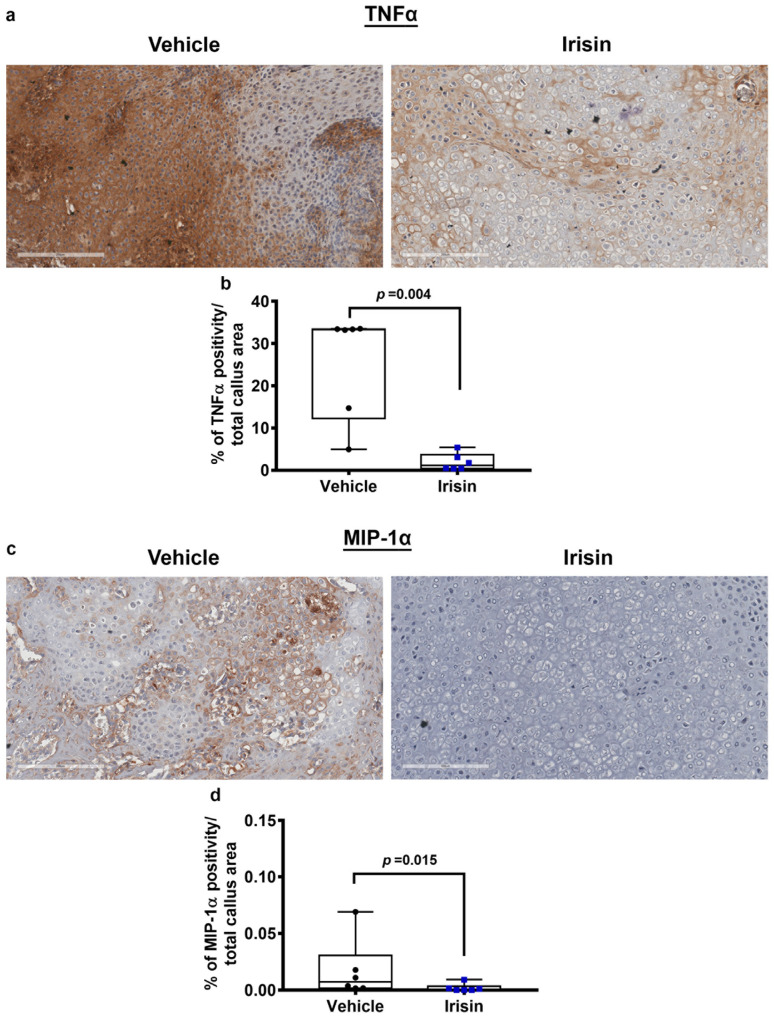
Representative images of (**a**) TNFα and (**c**) MIP-1α immunostaining on callus section obtained from vehicle-treated mice (*n* = 6) and irisin-treated mice (*n* = 6) at 10 days after fracture (scale bars 200 µm). Dot plots showing quantification of (**b**) TNFα (**d**) and MIP-1α expression. Data are shown as dot plots with median, from max to min, with all data points shown. Mann–Whitney test was used to compare groups.

To evaluate the influence of irisin treatment on TNFα levels during fracture repair, immunohistochemistry was performed on soft callus sections obtained at 10 days post fracture. Immunostaining for TNFα (Figure 2a) and relative quantification (Figure 2b), revealed a significant decrease (*p* = 0.004) of TNFα expression in the soft callus of irisin-treated mice compared with the vehicle group, suggesting that irisin promotes TNFα decrease at 10 days post fracture.

Furthermore, the expression of the pro-inflammatory cytokine MIP-1α within the callus tissue was assessed by immunohistochemistry (Figure 2c), and its quantification showed a significant decrease (*p* = 0.015) at 10 days after fracture in irisin-treated mice compared with controls (Figure 2d), thus suggesting an inhibitory effect of irisin on MIP-1α expression levels on soft callus.

### 2.3. Irisin Promotes Angiogenesis and Matrix Degradation at 10 Days Post Fracture

**Figure 3:**
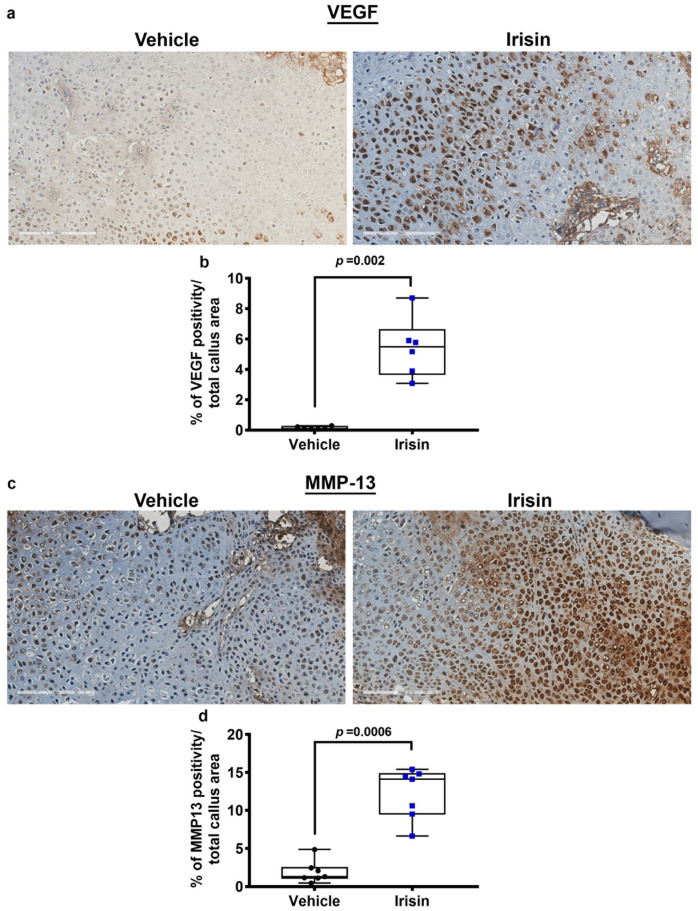
Representative images of (**a**) VEGF and (**c**) MMP-13 immunostaining on callus section from vehicle-treated mice (*n* = 6) and irisin-treated mice (*n* = 6) 10 days after fracture (scale bars 200 µm). Quantification of (**b**) VEGF and (**d**) MMP-13 expression are shown as dot plots. Data are presented as dot plots with median, from max to min, and all data points are shown. Mann–Whitney test was used to compare groups.

Immunohistochemical staining for the angiogenic molecule VEGF on callus sections obtained at 10 days post fracture (Figure 3a) and relative quantification (Figure 3b) showed a 40-fold increase (*p* = 0.002) in the callus of irisin-treated mice compared to the vehicle group.

In parallel, MMP-13, a metalloproteinase necessary for cartilage degradation and vascular invasion, was also significantly higher (*p* = 0.0006) in the callus of irisin-treated mice than in the vehicle-treated group (Figure 3c,d).

### 2.4. Irisin Induces the Secretion of an Osteogenic Factor at 10 Days Post-Fracture

**Figure 4:**
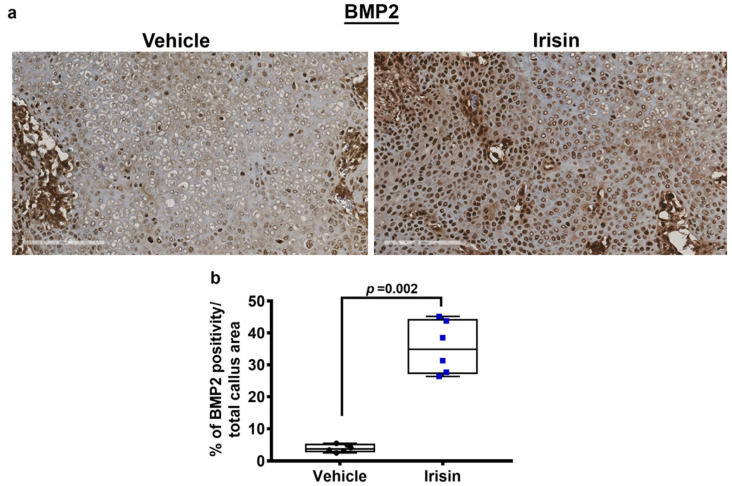
Representative images of (**a**) BMP2 immunostaining on callus section from vehicle-treated mice (*n* = 6) and irisin-treated mice (*n* = 6) 10 days after fracture (scale bars 200 µm). Quantification of (**b**) BMP2 expression is shown as dot plots. Data are presented as dot plots with median, from max to min, and all data points are shown. Mann–Whitney test was used to compare groups.

Immunostaining for BMP2, an osteoinductive molecule involved in the differentiation of mesenchymal progenitor into chondrocytes or osteoblasts, revealed a nine-fold increase (*p* = 0.002) of BMP2 expression levels in the soft callus of irisin-treated mice compared with the vehicle group (Figure 4a,b), suggesting an important role of irisin in mediating fracture repair.

## 3. Discussion

Fracture healing involves the spatiotemporal succession of precise events mediated by the secretion of molecules differently expressed at various stages of the process. Recent data demonstrated an important role of the myokine irisin in both modulating the gene expression pattern of chondrocytes towards the hypertrophic phenotype and accelerating fracture repair in mice by inducing maturation of the soft and bony callus [34]. The effect of irisin on fracture repair is mainly achieved through enhanced coupling of osteogenesis [34] and angiogenesis [35]. Here, we report that the involvement of irisin in promoting soft callus formation in mice at 10 days post fracture could also be related to its ability to synchronously modulate the expression of inflammatory, angiogenic, and osteogenic molecules.

Firstly, we evaluated whether irisin could modulate the expression levels of the pro-inflammatory cytokine TNFα, as previous preclinical studies showed a potent inhibitory effect of irisin on its expression in a rat model of inflammatory bowel disease [36]. We observed a decrease in TNFα expression levels in irisin-injected mice compared with the control group on day 10 after tibia fracture. TNFα, produced by resident macrophages and recruited inflammatory cells, represents a primary mediator in the inflammation process during the initial phase of the fracture healing while returning to baseline levels during the following period for most of the remaining reparative process. Variation from this temporal distribution of this molecule within the fracture site could negatively affect fracture healing [37,38]. Therefore, the ability of irisin to reduce TNFα levels during fracture repair could contribute to accelerating the cartilage fracture callus production, thus reducing the time required for soft callus maturation. This observation is in line with previous data demonstrating that irisin promotes bone fracture healing at a faster rate than vehicle treatment. Noteworthy, we previously highlighted the involvement of irisin in increasing the expression levels of the osteoblastic differentiation marker Runt-related transcription factor 2 (Runx2) at 10 days after fracture, thus inducing the transition of cartilaginous callus into bony callus. Consistent with this finding, other authors observed that, 8 days after fracture, the inhibition of TNFα stimulated Runx2 expression and promoted trabecular bone formation at the fracture site [8], further supporting the importance of irisin in modulating TNFα levels in our mouse model of fracture healing.

The efficacy of irisin in blocking inflammation events at the stage when the cartilaginous callus should progress toward ossification is also due to its ability in reducing the expression levels of MIP-1α, another well-known inflammatory cytokine produced by macrophages. Previous studies have shown that inflammatory protein MIP-1α expression in the fracture callus was upregulated in the first 3 days after fracture in both animal [39] and human models [40]. However, in a diabetic rat model with systemic inflammation, increased serum MIP-1α levels were also associated with delayed fracture healing [41]. In agreement with this, it is very relevant that irisin keeps the levels of this inflammatory chemokine lower to ensure timely fracture healing.

In addition, histological analysis of the fractured tibiae showed that irisin strongly increased the expression of the well-known pro-angiogenic factor VEGF, in agreement with Kan and colleagues [35], who found higher levels of this growth factor in the femurs of irisin-treated mice compared with the control group at 14- and 21-days post femur fracture. It is well known that during fracture healing, angiogenesis is essential during the stage of fibrocartilaginous soft callus formation and its subsequent transition into a bony callus. The invasion of vessels into the cartilaginous callus is necessary for the arrival of osteoblast precursors and the subsequent repair process [3]. Indeed, it has been shown that in Runx2 knockout mice, VEGF is not expressed in hypertrophic chondrocytes resulting in a lack of vascularization in the growth plate [42]. These data indicate that the vascularization in the growth plate is regulated by VEGF, produced by numerous different cell types, such as chondrocytes, hypertrophic chondrocytes, and osteoblasts [3]. Therefore, by assuming that an alteration in the process of angiogenesis leads to the impairment of the fracture healing process, the irisin-mediated increase in VEGF could represent an important new mechanism explaining the ability of the myokine to accelerate the bony callus formation.

It is also known that vessel invasion into the soft callus is mutually coupled with cartilage matrix degradation and bone formation during the cartilage-to-bone transition. Hypertrophic chondrocytes express MMP-13 [12], a metalloproteinase essential for cartilage degradation and vascularization into the soft callus as demonstrated by the expanded hypertrophic zone and a delay of vascularization in the growth plate of MMP-13-deficient mice [43]. Interestingly, we found that irisin treatment promotes an increase of MMP-13 levels in the fractured tibiae of mice, thus providing another possible mechanism through which irisin improves fracture healing.

Finally, we also demonstrated that irisin treatment strongly enhanced the expression of the osteogenic molecule BMP2, an essential mediator for fracture repair [10]. Kan et al. observed higher BMP2 expression levels in the irisin-treated group compared to the controls at 2 and 3 weeks post fracture [35]. Interestingly, we found that irisin-induced up-regulation of BMP2 occurred earlier in time (10 days post fracture), thus suggesting that the potent osteogenic stimulus provided by BMP2 already ensues when cartilage callus is still present. Accordingly, an elegant paper by Mi and colleagues investigated the role of endogenous BMP2 in chondrocytes and osteoblasts during fracture healing by analyzing chondrocyte-specific or osteoblast-specific BMP2 conditional knockout mice [44]. The authors showed that deletion of BMP2 in chondrocytes resulted in prolonged retention of the cartilaginous callus, with delayed osteogenesis and impaired biomechanical properties of the callus. In contrast, when the BMP2 gene was deleted in osteoblasts, no significant differences were observed in the fracture healing process compared with control mice [44]. Taken together, the results suggest that endogenous expression of BMP2 in chondrocytes, enhanced by irisin, as shown herein, could play a key role in cartilaginous callus maturation at an early stage of fracture healing.

Our study has some limitations. First, data were obtained only by histological and morphometric analysis. Second, our investigation was limited by the single time point of analysis (10 days post fracture).

Overall, our results demonstrate that systemic administration of a low intermittent dose of irisin in a mouse model of tibial fracture can accelerate the healing process by already acting at an early post-fracture stage given that this myokine might be able to simultaneously switch off inflammation, activate angiogenesis, and initiate the degradation, and thus replacement, of cartilaginous matrix. Future studies will be needed to understand whether local administration of irisin could have even more beneficial effects in terms of healing time and bone callus quality than systemic administration.

Consistent with the similar healing fracture processes that occur in mice and humans, [45] our data could be useful to further investigate the role of irisin as a promising new therapeutic strategy to improve and accelerate the process of fracture repair.

## 4. Materials and Methods

### 4.1. Tibial Fracture Procedure and Experimental Design

Eight-week-,old C57BL/6 male mice were subjected to closed, transverse, mid-diaphyseal tibial fractures on the right tibia [34]. After anesthesia with isofluorane gas, a small incision from the medial to the tibial tuberosity was made. Then, the bone cortex of tibial plateau was punctured using a 26-gauge needle, and a 0.22 mm sterile diameter pin was inserted through the length of the intramedullary canal. Fractures were generated using a custom guillotine device and the incision was closed with surgical sutures. Subcutaneous injection of buprenorphine (0.05 mg/kg) was performed pre and post operation, and carprofen (5 mg/kg) was injected immediately post operation and during the recovery period. To check fracture position and proper pin placement, post-surgery X-ray scans were generated using a microradiography system (Faxitron, Wheeling, IL, USA).

**Figure 5:**
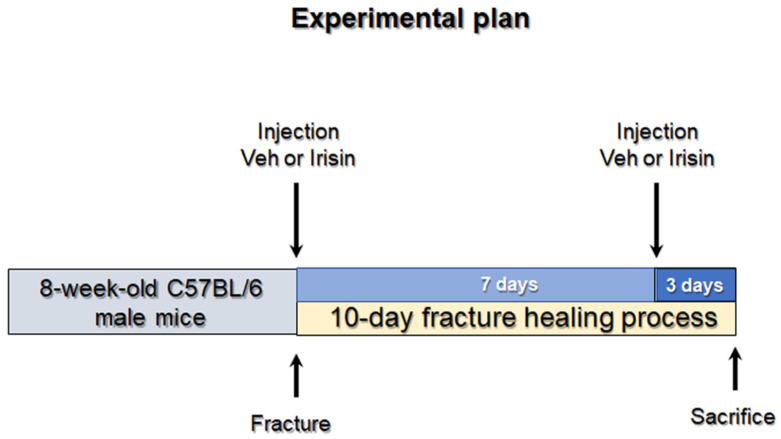
Experimental plan. Eight-week-old C57BL/6 male mice were subjected to closed, transverse, mid-diaphyseal tibial fractures on the right tibia. One group (*n* = 6 vehicle) was intra-peritoneally (i.p.) injected with phosphate buffer solution (PBS), and the other group (*n* = 6 irisin-treated mice) was injected with 100 mg/kg irisin at days 0 and 7. All mice were sacrificed 10 days post fracture induction.

Of 17 mice undergoing fracture induction, *n* = 5 mice with a displaced fracture were excluded from post-fracture treatment. Mice were randomly divided into two groups: one group (*n* = 6 vehicle) was intra-peritoneally (i.p.) injected with phosphate buffer solution (PBS) and the other group (*n* = 6 irisin-treated mice) was injected with 100 mg/kg irisin at days 0 and 7 (Figure 5). All mice were sacrificed 10 days post fracture induction. The injected myokine was untagged recombinant irisin produced in *E. coli* (Adipogen International, San Diego, CA, USA) previously validated by ELISA assay [17].

Mice were housed in rodent cages with access to water and regular chow diet ad libitum and maintained under standard conditions on a 12/12 h light/dark cycle (Harlan Teklad 2019, SDS, England). After the pre-established healing periods, euthanasia was performed, and bone segments were fixed 72 h in PFA 4%. All animal experiments described in this article were reviewed and approved by the University of Michigan’s Committee on Use and Care of Animals Protocol #PRO00008779 (Goldstein).

### 4.2. X-ray and Micro-Computed Tomography

Using the microradiography system Faxitron, X-ray scans were also taken immediately after euthanasia to observe callus conformation at 10 days. After removing the intramedullary pins, all fracture calluses were dissected from attached muscle and stored in 70% ethanol. The fractured tibiae were scanned using an eXplore Locus SP microCT system (GE Healthcare, London, ON, Canada). Scanning parameters included 80 kVp and 80 μA X-ray source, rotation angle with 0.5° increment and 1600-millisecond exposure. The tibiae were immersed in distilled water to reduce beam-hardening artifacts, and a 0.02-inch aluminum filter was used with an acrylic beam flattener around the tibiae. Images were reconstructed to an isotropic voxel size of 18 μm and calibrated using a hydroxyapatite phantom. Images were taken using Microview Software MicroView 2.5.0rc7 (Parallax Innovations, London, ON, Canada).

### 4.3. Histological and Immunohistochemical Assays

**Figure A1:**
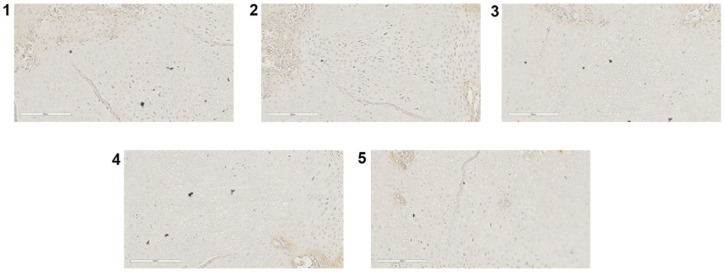
TNFα (**1**), MIP-1α (**2**), VEGF (**3**), MMP-13 (**4**), and BMP2 (**5**).

At 10 days, fractured tibiae were dissected and disarticulated from the knee, the surrounding muscles removed, then subjected to histomorphometric analysis. Fractured tibiae were decalcified with EDTA at 20% and pH 7.5, embedded in paraffin, and cut into 5 μm sections on a standard microtome (RM-2155 Leica, Heidelberg, Germany). H&E staining and immunohistochemistry were performed on sections collected from 10-day fractured tibiae from each mouse (vehicle, *n* = 6; irisin, *n* = 6) and using the Mouse and Rabbit Specific HRP Plus (ABC) Detection IHC Kit (ab93697 Abcam, Cambridge, United Kingdom). Sections were incubated with the following primary antibodies: Anti-TNFα Polyclonal Antibody (PA1-40281, Invitrogen) Anti-MIP-1α (ab259372, Abcam), Anti-VEGF Monoclonal Antibody (JH121, MA5-13182, Invitrogen), Anti-MMP-13 Monoclonal Antibody (VIIIA2, MA5-14238, Invitrogen) and Anti-BMP2 Policlonal Antibody (JH121, PA5-85956, Invitrogen). The same procedure was followed omitting the primary antibodies (negative controls), which are shown in Appendix A (Figure A1).

### 4.4. Morphometric Analysis

Stained sections were digitalized using the whole-slide scanning platform Aperio ScanScope CS (Leica Biosystems Nussloch, Germany) at the magnification of 20× and stored as high-resolution digital images on the workstation associated with the instrument. Morphometric analysis was performed by two independent observers on two adjacent selected sections from each side of each callus’s widest area. A total callus area of 13.5 mm^2^, corresponding to the widest callus area (region of interest, ROI) of all callus sections, was analyzed using the Aperio Color Deconvolution algorithm, enclosed in the ImageScope v.11.2.0.780 (Leica Biosystems). The algorithm allows us to distinguish the various colors (callus, and different IHC markers) of the stained tissue images converting them into digitalized separated channels. Then, each single-color channel was analyzed applying the Aperio Positive Pixel Count algorithm which was set to detect the number of strong positive pixels (Nsp), medium-positive pixels (Np), weak-positive pixels (Nwp) The percentage of total positive pixels was reported in graphs.

### 4.5. Statistical Analysis

All data are presented as boxplots with median, interquartile range, maximum, and minimum values. All variables were checked for normality (Shapiro–Wilk normality test) to see the data distribution. All parameters resulted as non-normally distributed; significance was evaluated with Mann–Whitney U test using GraphPad Prism (GraphPad Software, Inc. La Jolla, CA, USA). Differences were considered significant when *p* < 0.05.

## Data Availability

The data that support the findings of this study are available on request from the corresponding author.
